# Eugene P. Kennedy’s Legacy: Defining Bacterial Phospholipid Pathways and Function

**DOI:** 10.3389/fmolb.2021.666203

**Published:** 2021-03-25

**Authors:** William Dowhan, Mikhail Bogdanov

**Affiliations:** Department of Biochemistry and Molecular Biology, McGovern Medical School, University of Texas Health Science Center, Houston, TX, United States

**Keywords:** *Escherichia coli*, phospholipid metabolism, membrane proteins, Gram-negative, charge balance rule, lipid asymmetry, protein folding

## Abstract

In the 1950’s and 1960’s Eugene P. Kennedy laid out the blueprint for phospholipid biosynthesis in somatic cells and *Escherichia coli*, which have been coined the Kennedy Pathways for phospholipid biosynthesis. His research group continued to make seminal contributions in the area of phospholipids until his retirement in the early 1990’s. During these years he mentored many young scientists that continued to build on his early discoveries and who also mentored additional scientists that continue to make important contributions in areas related to phospholipids and membrane biogenesis. This review will focus on the initial *E. coli* Kennedy Pathways and how his early contributions have laid the foundation for our current understanding of bacterial phospholipid genetics, biochemistry and function as carried on by his scientific progeny and others who have been inspired to study microbial phospholipids.

## Introduction

Studies of lipid metabolism were first initiated in plants and mammals in the 1920 followed by the establishment of the Kennedy Pathway for phospholipid synthesis in mammalian cells in the 1950’s (Kennedy, 1992). It was not until the 1960’s that serious studies of bacterial lipid metabolism began largely in the laboratory of Eugene Kennedy at Harvard Medical School. During this decade Kennedy’s laboratory defined the pathways for the synthesis of the major phospholipids [phosphatidylethanolamine (PE), phosphatidylglycerol (PG) and cardiolipin (CL)] in *Escherichia coli*. With this blueprint in hand, the following two decades experienced an explosion in *E. coli* glycerol-based phospholipid metabolism, enzymology, genetics and function largely by Kennedy and his scientific progeny. Lipid A-base lipopolysaccharide (LPS) is the other phospholipid of Gram-negative that forms the outer lipid leaflet of the outer membrane of Gram-negative bacteria. The biochemistry, enzymology and genetics of Lipid A were largely defined by Chris Raetz (Dowhan et al., 2013; Whitfield and Trent, 2014), a trainee of the Kennedy lab. These initial findings laid the foundation for continuing studies in *E. coli* and other microbial systems including yeast providing excellent model systems for studying lipid involvement in bacterial antibiotic resistance and function of lipids in both prokaryotic and eukaryotic systems. The current state of pathways, enzymology, and genetics of glycerol-based phospholipid biosynthesis in *E. coli* will be reviewed followed by how this collective information has been used to establish specific functions for individual phospholipids in bacteria. Membrane bilayer phospholipid asymmetry studies are well advanced in eukaryotic cells but only beginning to be extensively studied in bacteria. Methods for determining and perturbing lipid bilayer asymmetry in Gram-negative bacteria will be reviewed as a prerequisite for determining the role of such asymmetry in cell function. Hopefully this review will provide a basis for extended studies of phospholipids in other bacteria and lay the foundation for further studies in *E. coli*.

## Current State of the Bacterial Kennedy Pathway

**FIGURE 1:**
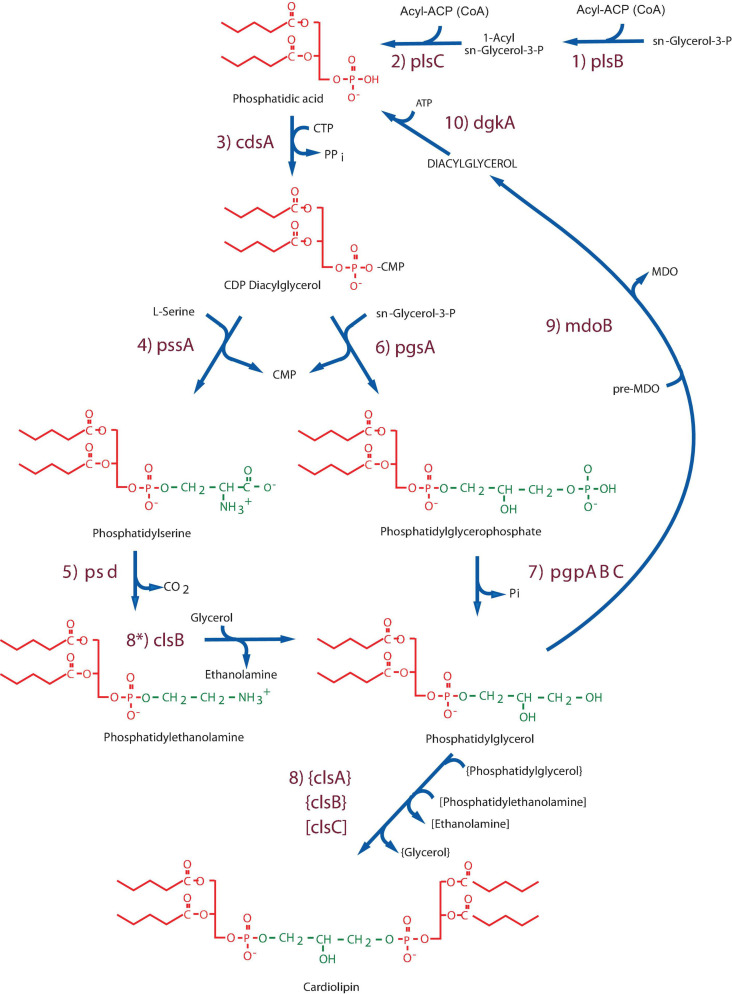
Kennedy Pathway for synthesis of phospholipids in *Escherichia coli*. The following enzymes with their respective genes named carry out: (1) G3P acyltransferase (PlsB); (2) 1-Acyl-G3P acyltransferase (PlsC); (3) CDP-DAG synthase (CdsA); (4) phosphatidylserine synthase (PssA); (5) phosphatidylserine decarboxylase (Psd); (6) phosphatidylglycerophosphate synthase (PgsA); (7) phosphatidylglycerophosphate phosphatases (PgpABC) encoded by three genes; (8) cardiolipin synthase (Cls) encoded by three genes. ClsA and ClsB condense 2 PGs while ClsC condenses PG and PE. ClsB (8*) can also displace ethanolamine from PE using glycerol to make PG, which can be utilized to make CL in *pgsA* null strains. (9) PG pre-membrane derived oligosaccharide (MDO) *sn*-glycerol-1-*P* transferase (MDO synthase); 10. DAG kinase (DgkA). Figure (modified) and legend reprinted by permission from Elsevier (Dowhan, 2013): Copyright 2013.

In *E. coli* (see Figure 1) and other γ-proteobacteria phosphatidic acid (PA) biosynthesis begins by acylation at the 1-position of *sn*-glycerol-3-phosphate (G3P) by either a long chain fatty acid- (primarily palmitic acid) acyl carrier protein (ACP) or CoA derivative catalyzed by PlsB (Ray et al., 1970). Many Gram-negative bacteria and all Gram-positive bacteria utilize PlsY (Lu et al., 2006), which use acyl-phosphate derivatives of long chain fatty acids. Acylation at the 2-position is catalyzed by PlsC (Coleman, 1990), which uses both ACP and CoA fatty acids (mainly long chain unsaturated) derivatives in γ-proteobacteria and only ACP derivatives in most other bacteria. The two-step acylation process generates membrane-residing PA followed by conversion of this short-lived intermediate precursor to CDP-diacylglycerol (CDP-DAG), which functions as a donor of phosphatidyl moieties to the primary hydroxyl groups of either L-serine or G3P to form phosphatidylserine (PS) or phosphatidylglycerol phosphate (PGP), respectively. The latter is dephosphorylated to form PG and the former is decarboxylated to form PE. Some of the PG pool is further converted to CL. PE, PG, and CL are the major end products of the Kennedy Pathway and the primary lipid components of the inner membrane and periplasmic leaflet of the outer membrane. The levels of the intermediates PA, CDP-DAG, PS, and PGP are extremely low in wild-type *E. coli*, representing less than 0.1–0.3% of the total cellular phospholipid. These intermediates are found in higher levels in *E. coli* lipid mutants as will be discussed below. In *E. coli* diacylglycerol (DAG) derived from transfer of *sn*-glycerol-1-phosphate from PG in the formation of membrane derived oligosaccharide (MDO) (Schneider et al., 1979; Goldberg et al., 1981) is phosphorylated by DgkA to generate PA in the inner membrane (Pieringer and Kunnes, 1965; Raetz and Newman, 1978). MDO is a periplasmic oligosaccharide osmoregulatory decorated by *sn*-glycerol-1-phosphate and ethanolamine-phosphate derived from PE (Kennedy et al., 1976). The gene for the latter decoration has not been identified. Interestingly, details of the synthesis of PA came well after most of the Kennedy Pathway was worked out for *E. coli*.

Several differences exist in phospholipid biosynthetic pathways between prokaryotes and eukaryotes and within prokaryotes. Two *de novo* biosynthetic routes, collectively also known as the Kennedy Pathway were elucidated over 60 years ago and are responsible for the production of the majority of phosphatidylcholine (PC) and PE in most eukaryotic cells (Kennedy, 1992). CDP-choline or CDP-ethanolamine is formed by condensation of CTP with choline-phosphate or ethanolamine-phosphate followed by reaction with DAG to form phosphatidylcholine (PC) or PE, respectively (Kennedy and Weiss, 1956). Although *E. coli* does not contain PC, many Gram-negative bacteria contain PC made either by methylation of PE or phosphatidyl transfer from CDP-DAG to choline (Lopez-Lara and Geiger, 2017).

Somatic cells synthesize PS by headgroup exchange between PE or PC with L-serine (Stone and Vance, 2000). Bacteria (Kanfer and Kennedy, 1962) and most fungi (Bae-Lee and Carman, 1984) utilize the prokaryotic pathway where L-serine displaces CMP from CDP-DAG to form PS. Psd, which is highly homologous between eukaryotes and prokaryotes (Voelker, 1997), catalyzes the decarboxylation of PS to PE (Kanfer and Kennedy, 1964). The decarboxylation pathway is the sole route for PE biosynthesis in *E. coli* and the major one in *Saccharomyces cerevisiae*.

A single PgsA catalyzes the displacement of CMP by G3P to form PGP in *E. coli* (Kanfer and Kennedy, 1964) and the mitochondria of somatic cells (Kiyasu et al., 1963; Kawasaki et al., 1999, 2001) and yeast (Chang et al., 1998). Eukaryotes utilize a single essential mitochondrial-localized PGP phosphatase (Zhang et al., 2011) while prokaryotes have multiple activities (Icho and Raetz, 1983; Funk et al., 1992) with PgpA (Chang and Kennedy, 1967) being the primary phosphatase. Most prokaryotes express multiple CL synthases with ClsA being the primary activity in *E. coli* with little understanding of the function of the other enzymes. ClsA is present under all growth conditions while ClsB and ClsC are induced during late log and stationary phases of growth (Tan et al., 2012). In bacteria synthesis of CL proceeds by a non-energy requiring condensation of two PG molecules with one PG acting as phosphatidyl donor and the other as a phosphatidyl acceptor with the release of glycerol (Hirschberg and Kennedy, 1972). Interestingly, ClsB also catalyzes headgroup exchange between PE and glycerol to form low amounts of PG and free ethanolamine (Li et al., 2016).

In mitochondria the formation of CL is energy dependent utilizing a transfer of PA from CDP-DAG to the terminal hydroxyl of PG (Hostetler et al., 1972). This irreversible reaction exhausts the pool of PG to maintain high CL and low PG levels in mitochondria. In contrast bacteria exhibit higher levels of PG versus CL. Since the bacterial energy independent reaction is fully reversible, the PG/CL ratio can change with an increase in CL levels in the stationary phase (Hiraoka et al., 1993) or after abiotic osmotic or thermal insults (Luevano-Martinez et al., 2015). The line between eukaryotic and prokaryotic Cls enzymes has become blurred by identification of a eukaryote-type CDP-DAG-dependent-Cls in *Streptomyces coelicolor* (Sandoval-Calderon et al., 2009) and a potentially PG condensing-Cls in the protozoan *Trypanosoma brucei* (Serricchio and Bütikofer, 2012).

The diversity of membrane lipid compositions within the Eubacterial (Lopez-Lara and Geiger, 2017) and Archaebacterial (Matsumi et al., 2011). Kingdoms is as diverse as the number of organisms; the latter phospholipids are characterized by containing *sn*-glycerol-1-phosphate in ether linkage to long chain isoprenoid alcohols. However, the pathways for generating the hydrophilic headgroups of the major phospholipids are very similar to that in Eubacteria. Covering this diversity is well beyond the scope of this review. The Kennedy Pathway phospholipid biosynthetic blueprint for *E. coli* has provided a starting point for elucidating lipid biosynthetic pathways throughout these kingdoms where differences and similarity to that of *E. coli* have been detailed.

## Genetics of Phospholipid Metabolism

All the genes encoding the pathways depicted in Figure 1 have been identified and their sequences are available either from direct sequencing or the sequence of the *E. coli* genome. Furthermore, there are mutants available for each gene. The first mutants reported in *E. coli* phospholipid synthesis were in PlsB (Bell, 1974, 1975) and PssA (Okonogi et al., 1971; Ohta et al., 1974; Ohta and Shibuya, 1977). However, a major advance in phospholipid biosynthetic enzyme genetics can be contributed to Chris Raetz, a disciple of the Kennedy lab. Since it was initially thought that null mutants in genes encoding these enzymes would be lethal, Raetz isolated conditionally lethal temperature sensitive mutants using a novel filter paper assay method (Raetz, 1975). Using this technique mutants were isolated in *pssA* (Raetz et al., 1977), *pgsA* (Raetz, 1975), *pgpAB* (Icho and Raetz, 1983; Icho, 1988a, b), *cdsA* (Ganong et al., 1980; Icho et al., 1985b) and *dgkA* (Raetz and Newman, 1978; Lightner et al., 1983).

A *psd* temperature sensitive mutant was isolated by a modified brute force scanning after mutagenesis (Hawrot and Kennedy, 1976). A glycerol auxotroph turned out to encode a PlsB mutant with a 10-fold higher Km for G3P (Bell, 1974, 1975; Lightner et al., 1980). A mutation in *mdoB* (Jackson et al., 1984) was isolated as a suppressor of the large accumulation of DAG in a *dgkA* mutant. A screen of temperature sensitive mutants that were sensitive to deoxycholate (indicative of a defect in outer membrane integrity) turned out to be mutants in *plsC* (Coleman, 1990). Null mutants in the *dgkA* gene result in a 20-fold accumulation of DAG, which in an osmotically challenging environments is lethal (Raetz and Newman, 1978) due to failure of cells to divide (Kawashima et al., 2008). Therefore, DgkA acts as a salvage enzyme to permit the reutilization of DAG molecules for phospholipid synthesis in wild-type cells (Kennedy, 1982). MDO biogenesis accounts for only 2/3 of the DAG that accumulates upon *dgkA* inactivation (Rotering and Raetz, 1983). Most likely the remaining DAG results from LPS modification by transfer of ethanolamine-phosphate from PE catalyzed by the *eptA* gene product (Reynolds et al., 2005; Samantha and Vrielink, 2020).

The *pgpAB* double null strain still contains PGP phosphatase activity and grows normally. The original screening for phosphatase mutants missed PgsC, which is temperature sensitive at 42°C (Funk et al., 1992). Screening for increased PGP phosphatase activity in the *pgpAB* double null mutant expressing a plasmid library of the *E. coli* genome led to identifying and cloning of the *pgpC* gene (Lu et al., 2011). A mutant in *clsA* was originally identified by brute force screening of strains defective in phospholipid metabolism (Pluschke et al., 1978; Ohta et al., 1985). Informatic analysis led to identifying two paralogs of ClsA and identifying of *clsB* (Guo and Tropp, 2000) and *clsC* (Tan et al., 2012).

With the availability of plasmid-borne genetic libraries of the *E. coli* genome and the sequence of the complete genome, the remainder of the genetic map of phospholipid metabolism became available. With some exceptions the isolation of mutants and subsequent cloning of the genes was mostly done by Kennedy and his scientific descendants.

## The Enzymes of Phospholipid Synthesis

Most of the enzymes encoded by the genes listed in Figure 1, with exception of PlsC, PgpAC, ClsB, ClsC, and MdoB, have been purified to near homogeneity. Once the respective genes were cloned and expressed from multi-copy plasmids, sufficient amounts of purified enzymes were available for mechanistic and structural studies. All enzymes in the pathway except PssA are integral membrane proteins and are associated with the inner membrane of *E. coli*. The combination of the genetic and biochemical studies has validation of the Kennedy Pathway at the molecular level.

### Synthesis of PA

Purification of PlsB to near homogeneity (Larson et al., 1980; Green et al., 1981) was facilitate using *E. coli* strains carrying plasmids overexpressing the *plsB* gene (Clark et al., 1980). Sequencing of isolated clones revealed the amino acid sequence of PlsB (91,381 Da). PlsC (27, 500 Da) has been partially purified (Coleman, 1992) and from informatic analysis of its sequence, PlsC appears to be similar to the catalytic properties of PlsB (Coleman, 1990; Yao and Rock, 2013). Although saturated and unsaturated long chain fatty acids are substrates, PlsB favors the former while PlsC favors the latter consistent with the acyl chain species of phospholipids found in *E. coli* (Goelz and Cronan, 1980). Orthologs of PlsB are found in several γ-proteobacteria. For more information on bacterial fatty acid synthesis (see Rock and Jackowski, 2002). PlsB and PlsC produce *de novo* PA while DgkA (13,245 Da) (Lightner et al., 1983) functions in recycling DAG generated in the synthesis of MDO. Crystal (Li et al., 2013) and NMR (Van Horn et al., 2009) structures of DgkA are available.

### CDP-DAG Synthase

CdsA catalyzes the activation of PA with CTP to generate CDP-DAG which serves as a precursor at the branch point of the Kennedy Pathway for the formation of zwitterionic PE and anionic PG plus CL. Cloning of the *cdsA* gene and expression from multicopy plasmids (Icho et al., 1985b) facilitated the purification of the synthase (27,570 Da) (Sparrow and Raetz, 1985). The enzyme utilizes equally only dCTP and CTP, favors PA with at least one unsaturated fatty and requires a divalent metal ion for activity. Like many integral membrane enzymes, purified CdsA requires its lipid substrate to be dispersed in detergent micelles and exhibits substrate dilution kinetics (Warner and Dennis, 1975; Carman et al., 1995). At a fixed lipid substrate concentration, the activity increases with increasing detergent concentration until all substrate is integrated into detergent micelles. Continued increase in detergent results in progressive decrease in apparent activity due to dilution of the substrate in the surface of the micelle. CdsA has affinity for detergent-phospholipid mixed micelles and once incorporated its activity is dependent on the mole fraction of substrate within the mixed micelle. The enzyme does not catalyze either CDP-diacylglycerol hydrolase activity or exchange activity between substrates and products. Bacterial and eukaryotic synthases display significant homology throughout nature (Dowhan, 1997a), which was used to clone the respective synthase genes encoding endoplasmic reticulum Cds1 (Shen et al., 1996) and mitochondrial Tam41 (Tamura et al., 2013) from *S. cerevisiae*, and the *Drosophila* (Wu et al., 1995) and human (Weeks et al., 1997) genes.

### PS Synthase

PssA’s in Gram-negative bacteria including *E. coli* (Raetz and Kennedy, 1972, 1974) are unique in that they are not associated with the membrane in cell free extracts but are tightly associated with ribosomes (Dutt and Dowhan, 1977). In *Bacillus subtilis* (Okada et al., 1994), *Bacillus licheniformis* (Dutt and Dowhan, 1981) and *S. cerevisiae* (Bae-Lee and Carman, 1984), the enzyme is an integral membrane protein requiring a divalent cation for activity, which the *E. coli* enzyme does not. The *B. subtilis* enzyme can fully substitute for the *E. coli* enzyme when expressed in a *pssA* null strain (Okada et al., 1994). The *E. coli* enzyme has been functionally expressed in wild type *B. subtilis* (Zhang et al., 2009), but it is not known whether it can substitute for native enzyme.

The affinity of *E. coli* PssA for ribosomes may not be physiological (Louie et al., 1986; Louie and Dowhan, 1980). Both termini sequences of PssA (52,817 Da) are enriched in positively charged amino acids, which may explain its strong affinity for polyphosphate surfaces such as the ribosome (DeChavigny et al., 1991). Although dissociation from the ribosomal fraction requires 5 M NaCl (Raetz and Kennedy, 1974), physiological levels of polyamines such as spermidine or mixed micelles of detergent plus the CDP-diacylglycerol substrate are sufficient to dissociate the enzyme from ribosomes. *E. coli* membranes enriched in CDP-diacylglycerol or PG result in transfer of the enzyme from the ribosomal to the membrane fraction in cell lysates. Interestingly, the association with PG is ionic being prevented by high ionic strength buffers while the association with lipid substrate is insensitive to salt levels suggesting physiological importance of different modes of membrane association and existence of potential feedback mechanism to provide anionic and zwitterionic membrane content homeostasis as discussed latter.

The affinity for polyphosphate surfaces was capitalized in the purification of the enzyme. The enzyme was first bound to phosphocellulose and specifically eluted using mixed micelles of detergent and lipid substrate (Larson and Dowhan, 1976). This affinity purification method coupled with enzyme overproduction using high copy number plasmids carrying the *pssA* gene (Raetz et al., 1977; Ohta et al., 1981a) made available increased amounts of enzyme for study.

Although PssA presents as a “soluble” enzyme, it aggregates in the absence of detergents. and as noted above, has high affinity for its membrane associated substrate even in the presence of ribosomes. This is consistent with its requirement for a lipid substrate-detergent mixed micelle and display of substrate dilution kinetics (Carman et al., 1995). PssA follows a Ping-Pong reaction mechanism (Raetz and Kennedy, 1974; Larson and Dowhan, 1976) with retention of configuration at the PA-linked phosphate in the CDP-DAG substrate indicating that the reaction path proceeds through a substrate-enzyme covalent intermediate (Raetz et al., 1987). This mechanism is also consistent with low hydrolase activity toward its lipid substrate and product and very low transfer rates of the PA moiety of CDP-DAG to glycerol and G3P.

Pss enzymes belong to two different families: type I (non-integral membrane form) in the phospholipase D-like family and type II (integral membrane form) in the CDP-alcohol phosphotransferase family (Sohlenkamp et al., 2004). PssA from *E. coli* is a type I enzyme whereas the integral membrane associated Pss enzymes from *Bacillus* and *S. cerevisiae* are type II enzymes. The yeast enzyme shows no homology with the bacterial enzymes. However, the *B. licheniformis* (Dutt and Dowhan, 1981) and yeast enzymes exhibit sequential ordered Bi–Bi kinetics with no hydrolase activities. In fact, the yeast enzyme proceeds with inversion of configuration at the PA-linked phosphate of the CDP-DAG substrate (Raetz et al., 1987) consistent with a Bi-Bi mechanism in which the formation and release of CMP from CDP–DAG is dependent on L-serine.

### PS Decarboxylase

Enzymes in phospholipid biosynthetic pathways are in very low amounts requiring several thousand-fold purifications from wild type cells. The first enzyme in the *E. coli* pathway to be purified was Psd (Dowhan et al., 1974), which was at the time among one of the few functional integral membrane proteins available in purified form. This purification demonstrated that it was possible to isolate functional phospholipid biosynthetic enzymes, which was followed in the next few years in other bacteria, yeast and somatic cells. The *E. coli* enzyme is a heterodimer derived from a proenzyme through autocatalytic serinolysis at Ser-254 resulting in two subunits of 28,579 and 7332 Da with the latter subunit containing an amino-terminus blocked by pyruvate (Li and Dowhan, 1988, 1990). During catalysis a Schiff’s base is formed between the L-serine α-amino group of PS and the pyruvate prosthetic group followed by decarboxylation. The crystal structure of the enzyme shows a dimer of the heterodimer with the hydrophobic N-terminal α-helices most likely inserted into the lipid bilayer (Watanabe et al., 2020). This face of the dimer displays a lipid substrate binding pocket into which covalently bound PE was resolved after reduction of the substrate-enzyme Schiff’s base. Thus far all Psd’s in nature are pyruvate-dependent enzymes (Voelker, 1997). The *B. subtilis* enzyme (Matsumoto et al., 1998) and the mitochondrial somatic cell (Kuge et al., 1991, 1996) and *S. cerevisiae* (Trotter et al., 1993) enzymes show significant homology to the *E. coli* enzyme, although yeast also contains a divergent Psd localized to the Golgi/vacuole (Trotter and Voelker, 1995). *E. coli* Psd exhibits substrate dilution kinetics (Warner and Dennis, 1975; Carman et al., 1995). The enzyme is specific for the diacyl and G3P back bone as well as the L-serine headgroup (Dowhan et al., 1974). Psd’s are very efficient in converting PS into PE resulting in levels of 0.1% or less of total phospholipid in membranes containing a Psd. In eukaryotic cells PS is synthesized in the endoplasmic reticulum from where it is trafficked to other cell membranes where levels can reach 10% except in the mitochondria where Psd completely converts PS to PE (Voelker, 1997).

### PGP Synthase

All PgsA’s described so far belong to the CDP-alcohol phosphotransferase family. A gene encoding a putative PgsA is present in almost all bacterial genomes, but there are and will be increasing exceptions (Makarova et al., 2001; Radka et al., 2020). The enzyme was initially purified from wild type *E. coli* using a novel CDP-DAG affinity column (Hirabayashi et al., 1976). Once the cloned and sequenced *pgsA* gene was available, overproduction of the enzyme (20,701 Da) facilitated purification in high amounts (Ohta et al., 1981b; Gopalakrishnan et al., 1986). Functionally, the enzyme is similar to *B. subtilis* and yeast Pss in that it is an integral membrane protein, requires a divalent metal ion for activity and proceeds via a sequential Bi-Bi mechanism. The homologous somatic cell PgsA, when engineered for expression in *E. coli*, exhibits overproduction of synthase activity (Kawasaki et al., 1999), although it is unknown whether it suppresses a *pgsA* null strain.

### PGP Phosphatases

The three phosphatase of *E. coli*, PgpA (19,400 Da) (Icho and Raetz, 1983; Icho, 1988a), PgpB (29,021 Da) (Icho and Raetz, 1983; Icho et al., 1985a), and PgpC (24,439 Da) (Funk et al., 1992; Lu et al., 2011) show no sequence homology, all dephosphorylate PGP efficiently and have different specificities toward other phosphorylated lipids. A triple *pgpABC* null mutant is not viable, demonstrating that no additional PGPs exist in wild type *E. coli.* PgpAC are specific for PGP and require Mg^2+^ for active. PgpB is divalent metal ion independent and possesses a broad substrate spectrum as shown by its capacity to dephosphorylate PGP, PA, lyso-PA, undecaprenyl pyrophosphate (C55-PP) (Touze et al., 2008) and DAG-pyrophosphate (Dillon et al., 1996). In contrast to PGP hydrolysis, which relies on a His/Asp/His catalytic triad of PgpB, the mechanism of C55-PP hydrolysis only requires the His/Asp diad (Tian et al., 2020). Potential orthologs of the three phosphatases are found throughout other bacteria, but they are not homologous to any eukaryotic lipid phosphatases.

PgpB has been purified to near homogeneity (Touze et al., 2008). High resolution crystal structures have been determined for PgpB, which detail catalytically important residues (Fan et al., 2014) and a PE binding site that stabilizes the enzyme (Tong et al., 2016). Strict concentration dependence of activity on PE may allow PgpB activity to remain at a physiologically required threshold providing an attractive mechanism on how membrane proteins achieve two competing requirements, stability and flexibility both required for function (Guo et al., 2020).

### CL Synthases

The bacterial CL synthases were originally thought to use CDP-DAG as substrate until definitive evidence confirmed that condensation of two PG molecules is catalyzed at least for what we know to be ClsA (Hirschberg and Kennedy, 1972). ClsA is the primary source of CL during exponential growth. The *clsA* gene was cloned, the enzyme overproduced and partially purified (Ohta et al., 1985; Hiraoka et al., 1991). The enzyme appears to follow substrate dilution kinetics (Hiraoka et al., 1991). Although the *clsA* DNA sequence indicates a protein of 54,822 Da, the mature protein is about 46,000 Da (Hiraoka et al., 1991; Quigley and Tropp, 2009). There appears to be a posttranslational shortening of the protein. High overproduction of the enzyme is lethal due to compromised cell membrane barrier function presumable due to overproduction of CL (Hiraoka et al., 1991). Interestingly, the active site of ClsA faces the periplasm of *E. coli* (Shibuya et al., 1985).

ClsB (47,634 Da) and ClsC (53,666 Da) were first identified as products of genes *ybhO* and *ymdC*, respectively, which encode proteins with high homology to ClsA (Guo and Tropp, 2000). *In vivo* studies originally missed the presence of ClsBC because these activities contribute low amounts to the CL pool and only at late exponential to stationary phases of growth (Tan et al., 2012). ClsAB both condense two molecules of PG to form CL, but ClsC transfers a phosphatidyl moiety from PE to PG (Tan et al., 2012). Clones of *clsC* containing the adjacent *ymdB* gene encode increased levels of ClsC by an unknown mechanism. Although a *clsABC* null strain contains no detectible CL, a *pgsA* null strain contains trace amounts of CL. It turns out that ClsB also replaces ethanolamine in PE with glycerol to make PG, which then is converted to CL (Tan et al., 2012).

Many questions remain unanswered with regards to CL synthesis in bacteria. Why are there multiple synthases and what are their function? What is the mechanism of *ymdB* amplification of ClsC? What is the topological orientation of the active sites of ClsBC and how are the membrane orientation of the three synthases related to their function? Is the ability of ClsB to make PG from PE of physiological significance?

## Which Enzyme Activities Are Essential?

Determining “essential” lipid genes is dependent on growth conditions. In the longer term evolution has selected genes necessary for optimal growth under a variety of conditions. Laboratory strains allow the identification of genes that are required for normal function but can be eliminated under artificial conditions leading to the identification of lipid dependent functions. No conditions have been identified to suppress the lethality of null mutants in genes encoding enzymes prior to and including the synthesis of CDP-DAG. Interestingly, growth conditions or secondary suppressor mutations have been identified to support null mutations in genes beyond the branch point. Therefore, *E. coli* stains are available completely lacking either PE and PS (DeChavigny et al., 1991), PG and CL (Asai et al., 1989; Kikuchi et al., 2000) or CL (Tan et al., 2012). However, no conditions have been found to support growth of strains lacking simultaneously PE, PS, and CL (DeChavigny et al., 1991).

The first null mutant in phospholipid synthesis in *E. coli* resulted from the interruption of the *pgsA* gene (Heacock and Dowhan, 1987, 1989). Lethality was suppressed by a plasmid copy of *pgsA* or a chromosomal copy of *pgsA* under *lacOP* promoter control in the presence its inducer (Heacock and Dowhan, 1989). Although *pgsA* is absolutely required in a wild type strain, transfer of the null allele into several genetic backgrounds had little effect on growth. The lethal effect in wild type strains is due to a requirement for PG as a DAG donor to the N-terminal cysteine thiol group of the major outer membrane lipoprotein (Lpp) in a reaction catalyzed by prolipoprotein DAG transferase (Lgt) (Asai et al., 1989; Kikuchi et al., 2000). This modification occurs in the inner membrane and is required prior to translocation of Lpp to the outer membrane. Accumulation of the precursor results in disruption of the inner membrane and cell lysis. A double null *pgsA lpp* strain still grows poorly at 37°C and lyses at 42°C. There are several other lipoproteins requiring the same modification whose defective maturation results in membrane stress and induction of the two-component Rcs phosphorelay signal transduction system making cells thermosensitive. Disruption of the *rcsA* gene suppresses the poor growth and temperature sensitivity of the *pgsA lpp* null strain (Nishijima et al., 1981; Shiba et al., 2004; Nagahama et al., 2006; Shiba et al., 2012). Therefore, in this complex suppressor strain PG and CL are not required for near normal growth. However, there are still several phenotypes of strains completely lacking PG and CL as discussed below.

The *pgsA* null viable strains still contain about 10% anionic phospholipids mainly PA, CDP-DAG and *N*-acyl-PE (Mileykovskaya et al., 2009). Accumulated PA and CDP-DAG in the mutant can also serve as DAG donors for thiol modification of lipoprotein precursors but only inefficiently (Tao et al., 2012). Therefore, these negatively charged lipids can also compensate for loss of PG and CL so one cannot conclude that there is no requirement for anionic phospholipids in *E. coli*.

As noted earlier, a *pgpABC* null mutant is not viable unless covered by a plasmid expressing one of the phosphatase genes (Lu et al., 2011). This is probably due to accumulation of PGP in the membrane since feeding a phosphonate analog of G3P to *E. coli*, which results in the accumulation of the non-hydrolysable phosphonate analog of PGP, is bacteriostatic (Tyhach et al., 1976).

Interruption of the *pssA* gene prevents the synthesis of PS and PE thus the strains lack all amino-containing and zwitterionic phospholipids (DeChavigny et al., 1991). PS is barely detectible in *E. coli*, so PE is the major zwitterionic net neutral lipid making the cell membranes highly anionic in its absence. Null *pssA* strains are viable in media containing 10–50 mmolar divalent cations Ca^2+^, Mg^2+^, or Sr^2+^, but not Ba^2+^ (DeChavigny et al., 1991; Rietveld et al., 1993, 1994; Killian et al., 1994) consistent with earlier reports of divalent metal ion suppression of temperature sensitive mutations in *pssA* (Raetz, 1976; Ohta and Shibuya, 1977) and *psd* (Hawrot and Kennedy, 1978). Removal of divalent cations from the growth medium results in rapid lysis. Null mutants require supplementation of minimal defined medium with all the amino acids. Even under optimal conditions the null mutant growth rate is two- to three-times slower and the cells are filamentous with multiple genomes (DeChavigny et al., 1991; Mileykovskaya and Dowhan, 2000). Therefore, lack of PE is essential for normal cell viability.

Construction of strains with an interrupted *psd* gene was unsuccessful even though supplementation of medium with divalent cations suppressed the lethality of temperature sensitive mutants. An interrupted chromosomal copy of the *psd* gene could be rescued by a plasmid carrying the *psd* gene along with surrounding genes suggesting a polar effect of the interruption on expression of essential genes in this region of the chromosome (Li, 1989).

*Escherichia coli* has a marked redundancy in CL synthesis with none of three gene products being essential under laboratory conditions. However, as noted later CL-deficient strains display several deficiencies, which would limit their survival or vitality under stressed conditions.

## Functions of the Major Phospholipids Beyond Barrier Maintenance

Membrane associated processes account for half or more of all cellular functions. Membranes are complex structures composed primarily of proteins and lipids stabilized by dynamic cooperative non-covalent interactions. Historically, studies of membrane associated functions have focused on the protein component while ignoring the role of membrane lipids in defining function. A primary role for lipids in forming the permeability barrier of cells and organelles was first proposed by Ernest Overton in 1895 (Overton, 1895). However, a lipid bilayer as opposed to a protein barrier was not accepted until many years later (Danielli and Daśon, 1935; Robertson, 1957) but still envisioned a lipid bilayer covered with a protein layer on each side. The fluid mosaic model of membrane structure (Singer and Nicolson, 1972), where laterally mobile proteins are embedded in a sea of lipids, combined many studies on membrane structure and remains a widely accepted representation of membrane structure (Nicolson, 2014). Minor lipids as cellular signaling molecules gained recognition in the 1970s and 1980s (Horrobin et al., 1977; Hokin, 1985) and remain a heavily studied area. Interestingly, it was Kennedy that first reported the synthesis of a phosphorylated phosphatidylinositol, an important lipid signaling molecule, in brain that underwent rapid formation and dephosphorylation (Paulus and Kennedy, 1960). There are few strong examples of lipid signaling in *E. coli*. One example is the outer membrane phospholipase A that deacylates phospholipids in the LPS rich outer leaflet of the outer membrane. The generated free fatty acids act as second messages in the CoA form that prevent the degradation of a central enzyme (LipC) in Lipid A biosynthesis thus maintaining synthesis of LPS precursors (May and Silhavy, 2018). However, recognition that the major membrane lipids affect membrane protein function and affect cellular function is more recent. Lack of focus on the importance of membrane lipid composition as a factor in cell function is due to a protein centric view (Popot and Engelman, 2016) driven by the ability of detergents to substitute for lipids in supporting the function of many purified membrane proteins.

How can the function of lipids beyond providing a permeability barrier be determined? A classical genetic approach to defining multiple functions for the major membrane lipids presents several barriers. Since lipids are not encoded directly by genes, mutants must be made in biosynthetic pathways, which can result in cell death due to loss of barrier function before a specific function is recognized. Pleiotropic effects on many cellular processes, particularly in multi-organelle cells, due to buildup of precursors or lack of final products complicates interpretation. Lipids have neither inherent catalytic activity nor obvious functions in isolation. Since we still do not know how to translate the physical and chemical properties of a single or complex mixture of lipid(s) into *in vivo* function, *in vitro* studies are prone to many artifacts especially when employing idealized lipid mixtures. Fortunately, at least in bacteria, it has been possible to establish conditions to support the growth of strains completely lacking the major phospholipid classes as noted above for *E. coli*. These strains are still compromised for cell growth and show recognizable phenotypes that provide clues to important roles for these lipids in normal cell growth.

**FIGURE 2:**
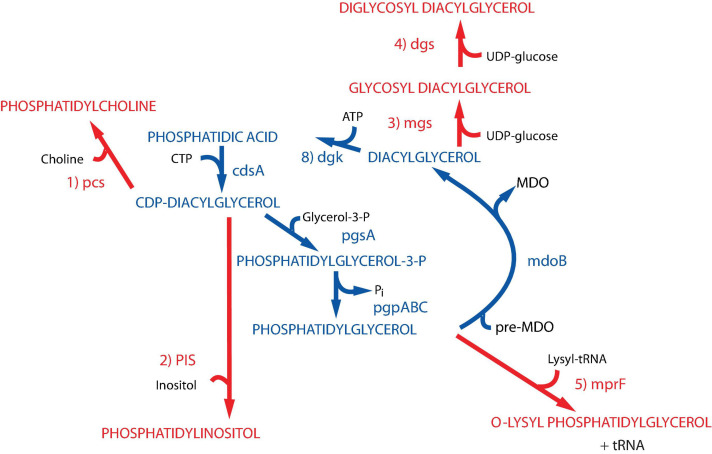
Synthesis of foreign lipids in *E. coli*. The native pathways in *E. coli* noted in *blue* are detailed in Figure 1. The enzymes with their respective genes named catalyze the following steps for synthesis of foreign phospholipids in *E. coli* noted in *red*: (1) phosphatidylcholine synthase [*Legionella pneumophila* (Conover et al., 2008; Bogdanov et al., 2010a)]; (2) phosphatidylinositol synthase [*Saccharomyces cerevisiae* (Xia and Dowhan, 1995b)]; (3) glucosyl diacylglycerol synthase [*Acholeplasma laidlawii* (Xie et al., 2006)]; (4) diglucosyl diacylglycerol synthase [*Acholeplasma laidlawii* (Wikström et al., 2009)]; (5) lysyl t-RNA phosphatidylglycerol lysine transferase [*Staphylococcus aureus* (Oku et al., 2004)]. Figure (modified) and legend reprinted by permission from Elsevier (Dowhan, 2013): Copyright 2013.

A set of viable *E. coli* “lipid mutants” (Figure 2) has been constructed in which native lipid composition can be systematically controlled at steady state (Wikström et al., 2004, 2009; Xie et al., 2006; Dowhan and Bogdanov, 2009; Bogdanov et al., 2010b), titrated in a dose-dependent manner (Bogdanov et al., 2008; Bogdanov and Dowhan, 2012; Dowhan, 2013) or varied temporally during the cell cycle (Zhang et al., 2003; Bogdanov et al., 2008, 2010a, b, 2014; Bogdanov and Dowhan, 2012). Lipids foreign to *E. coli* have been introduced as replacement of native lipids to understand the functional interchangeability and essential structural and charge properties of native lipids (Xia and Dowhan, 1995b; Wikström et al., 2004, 2009; Xie et al., 2006; Bogdanov et al., 2010a, b). Since membrane proteins and lipid environment have co-evolved, roles for the major lipids in membrane protein assembly and function only became evident when lipid composition was varied *in vivo*. *In vitro* biochemical characterization of the resulting phenotypes has differentiated direct from indirect effects of lipid-protein interactions and defined roles for lipids in membrane protein structure and function at the molecular level.

### Properties of Mutants Lacking PS and PE

Null *pssA* strains require mmolar levels of a select set of divalent cations and supplementation of minimal media with amino acids to support growth, display a filamentous growth phenotype, and are incompatible with a null *clsA* gene (DeChavigny et al., 1991). The requirement for a divalent cation and CL maybe related to the physical properties of the lipid bilayer. CL in the presence of a subset of divalent cations and PE are non-bilayer prone lipids due to their small hydrophilic headgroup versus their larger hydrophobic domain. This shape when present within a lipid bilayer causes local discontinuity and disruption, which appears to be universally required in natural bilayers. The minimum required concentration of divalent cations in the growth medium of a *pssA* null strain mirrors the strength of these ions to induce the non-bilayer phase for CL as follows: Ca^2+^ > Mg^2+^ > Sr^2+^ and Ba^2+^ is ineffective (DeChavigny et al., 1991; Rietveld et al., 1993, 1994; Killian et al., 1994). The temperature dependent mid-point of the transition from bilayer to non-bilayer phase for phospholipids extracted from wild type *E. coli* is about 55°C. The mid-point for phospholipids extracted from a *pssA* null strain grown under optimal concentrations of each of the above ions and suspended in the same concentration of ions is also 55°C. Phospholipids from cells grown in 10 mM Ca^2+^ have a lower CL content than those grown in 50 mM Mg^2+^ so when phospholipids extracted from cells grown in Ca^2+^ are suspended in Mg^2+^, the mid-point transition is well above 55°C. Conversely, when phospholipids extracted from cells grown in Mg^2+^ are suspended in Ca^2+^, the mid-point transition is well below 55°C. The growth dependence on Sr^2+^ shows a bell-shaped curve with a maximum at 12 mM, which is the same for the extracted phospholipids. The divalent cation requirement is extracellular since cytoplasmic Mg^2+^ levels are near 100 mM while Ca^2+^ levels are μM in *E. coli*. This close correlation between growth requirements and the phase properties of CL and PE strongly suggests a physical rather than chemical property of these lipids. Additionally, high monovalent or trivalent cations do not substitute for the divalent ions. The mechanism by which *E. coli* adjusts its CL level in apparent response to the divalent metal ion induced physical properties of CL is unknown. The fact that over production of CL by a plasmid borne copy of *clsA* under a non-native promoter results in cell lysis suggests the presence of some mechanism to regulate the physical properties of the membrane via the level of CL (Hiraoka et al., 1991).

Wild type *E. coli* do not require amino acids for growth and can utilize lactose as an energy source at μmolar levels. However, *pssA* null strains require mmolar levels of lactose as an energy source and all the amino acids. These requirements are due to mis-folding, as discussed in more detail later, of secondary transporters, which couple substrate accumulation to the proton electrochemical potential, for probably all amino acids and as well as lactose (DeChavigny et al., 1991; Zhang et al., 2003, 2005; Hariharan et al., 2018; Vitrac et al., 2020). It had been well known that PE is required for reconstitution of energy dependent uphill transport of substrate by the secondary transporter lactose permease (LacY) in proteoliposomes (Newman and Wilson, 1980; Newman et al., 1981). Cells lacking PE still transport lactose by energy independent downhill facilitated transport but cannot accumulate lactose against a concentration gradient due to a loss of coupling of transport to the proton electrochemical gradient, which is unaffected in *pssA* null strains (Bogdanov and Dowhan, 1995). Therefore, *pssA* null strains require higher levels of lactose in the growth medium to support growth. Similarly, in *pssA* null strains secondary amino acid transporters become facilitated transporters, which allows equilibration of endogenously synthetized amino acids with the growth medium thus reducing internal levels below that required to maintain growth. Primary transporters that utilize direct phosphorylation of substrates to achieve accumulation of substrate appear to be less affected in *pssA* null strains.

Filamentous growth is common among many mutations in membrane related processes. Null *pssA* mutants organize early cell division proteins at the FtsZ ring within multiple genomes but appear to have lost synchrony between cell growth and a late stage of cell division prior to constriction (Mileykovskaya et al., 1998). It is interesting that PE movement is required from the inner to the outer leaflet of the plasma membrane at the septum of yeast (Iwamoto et al., 2004) and somatic cells (Emoto and Umeda, 2000) prior to cell division followed by movement back to the inner leaflet during cell division. This suggests a universal requirement for PE in cell division.

### PE Acts as a Lipochaperone in a Late Stage of Membrane Protein Folding

**FIGURE 3:**
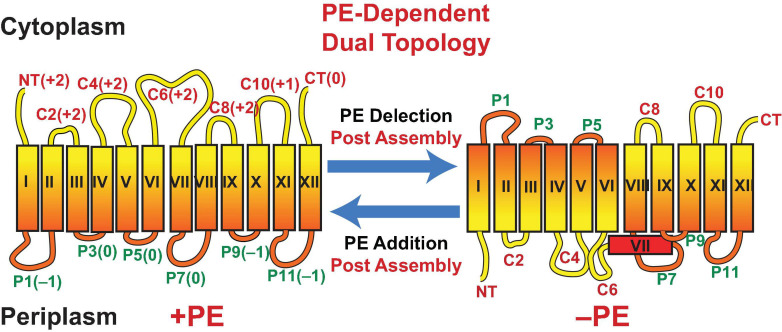
Topological organization of LacY as a function of membrane lipid composition. TMDs (Roman numerals) and EMDs (Arabic numerals) are sequentially numbered from the N-terminus to C-terminus with EMDs exposed to the periplasm (P) or cytoplasm (C) as in wild type cells. Net charge of EMDs is shown. Topology of LacY is shown after initial assembly in PE-containing cells (+PE) or after initial assembly in PE-lacking cells (–PE). The interconversion of topological conformers and the ratio of native to inverted conformer are reversible in both directions depending on the dynamic level of PE in membranes. Figure was modified and legend reprinted by permission from Springer Nature (Dowhan et al., 2017): Copyright 2017.

Assisting late stage folding of proteins has been restricted to protein chaperones. However, the misfolding of proteins in *E. coli* lacking PE demonstrated that lipids also act as chaperones (Bogdanov et al., 1996, 1999; Bogdanov and Dowhan, 1998, 1999). LacY from wild type cells maintains sufficient conformational memory even after SDS PAGE, which completely delipidates LacY, to be recognized by monoclonal antibody (mAb) 4B1; the 4B1 epitope lies within extramembrane domain (EMD) P7 of LacY (Figure 3) (Sun et al., 1996). However, LacY from PE-lacking cells is not recognized by mAb4B1 but is still recognized by mAb4B11, which recognizes native and denatured LacY; the 4B11 epitope is comprised of EMDs C8 and C10 of LacY (Sun et al., 1997). However, renaturation of LacY from *pssA* null cells after SDS PAGE in the presence of PE re-established recognition by mAb4B1. This was accomplished using the Eastern–Western technique where proteins separated by SDS PAGE are blotted onto a solid support layered with a test phospholipid. As SDS is electrophoresed away in the presence of PE, renaturation occurs as evidenced by recognition by mAb4B1. PE containing at least one saturated fatty acid (the predominant species in *E. coli*) but not PS restored recognition by mAb4B1; this is consistent with lack of uphill transport of lactose at the restrictive temperature in a *psd2* temperature sensitive mutant (Hawrot and Kennedy, 1978), which accumulates PS in the place of PE. Later it was found that PC containing at least one saturated fatty acid and neutral glycolipids (Figure 2) restored wild type conformation and function to LacY (Vitrac et al., 2013b). The earlier *in vitro* report that PC did not support uphill transport function of LacY reconstituted into proteoliposomes was due to the use of PC species containing only unsaturated fatty acids. Expression of PC (Bogdanov et al., 2010a) or neutral glycolipids (Xie et al., 2006; Vitrac et al., 2013b) in *E. coli* in the absence of PE or reconstitution *in vitro* in these lipids supports full function of LacY. Further demonstration of the involvement of PE in a late stage folding event was confirmed by restoration of mAb4B1 recognition of LacY initially assembled in PE-lacking *E. coli* membrane vesicles following *in vitro* synthesis of PE in these vesicles (Bogdanov and Dowhan, 1998). Therefore, PE fulfills the requirement of a lipochaperone by being required for proper protein folding during a late stage of protein maturation and no longer required once the protein is properly folded. PE is recognized as a lipochaperone supporting the function of several membrane associated processes in somatic cells (Patel and Witt, 2017).

### Charge Balance Rule for Membrane Protein Assembly

What is the molecular basis for LacY mis-folding in cells lacking PE? Prior to determination of the atomic structure of LacY (Abramson et al., 2003; Kumar et al., 2018), the substituted cysteine accessibility method to determine TMD orientation (SCAM^TM^) (Bogdanov, 2017) was used to establish a low-resolution structure of LacY that revealed the number and orientation of transmembrane domains (TMDs) and EMDs (Kaback et al., 2001) as shown in Figure 3. In this method the exposure of single cysteine residues in an otherwise cysteine-less protein to a membrane impermeable sulfhydryl reagent is used to map EMDs on the outside of cell membranes, isolated membrane vesicles and proteoliposomes or facing the lumen after membrane disruption. In PE-lacking cells (Bogdanov et al., 2002) the N-terminal six TMD-helical bundle is inverted with respect to the plane of the membrane bilayer and the C-terminal five TMD- helical bundle (Figure 3). In addition, TMD VII, which is of low hydrophobicity due to two Asp residues, is exposed to the periplasm. Disruption of LacY structure in the vicinity of EMD P7 and retention of structure in EMDs C8 and C10 is consistent with the mAb studies. The ratio of topological conformers (Dowhan et al., 2019), which is fully reversible *in vivo* (Bogdanov and Dowhan, 2012; Bogdanov et al., 2008) and *in vitro* (Vitrac et al., 2013a, 2015) is dependent on PE levels at the time of initial membrane assembly and is proportional post-assembly to changes in PE levels. There is no rapid interconversion of conformers at a fixed PE level but rather a change in PE level post-assembly drives interconversion. Inversion in the absence of PE is prevented by position-independent increases in the positive charge of EMDs C1–C6 or increasing the hydrophobicity of low hydrophobic TMD VII (necessary hinge point between the C- and N-helical bundles). The hydrophobic block can be reversed by increasing the negative charge of EMDs C1–C6. Inversion is induced in the presence of PE by increasing the negative charge of EMDs C1-C6. Phosphorylation of EMD C6 induces inversion, which is reversed by dephosphorylation (Vitrac et al., 2017, 2019). This was the first evidence that phosphorylation of a membrane protein can change its topological organization and possibly its function post-assembly. TMD flipping in proteoliposomes occurs on a time scale of seconds without the aid of any other cellular component and is rapid enough to be physiologically significant (Vitrac et al., 2013a, 2017). Since changes in topology only require a change in membrane lipid composition or a change in the net charge of an EMD, such interconversions can occur in any cellular membrane throughout nature without requiring any additional cellular components.

The vast majority of polytopic membrane proteins insert into membranes according to Positive Inside Rule with net positively charged EMDs facing the cytoplasm (von Heijne, 1986, 1989, 1992; Nilsson and von Heijne, 1990). However, the Positive Inside Rule cannot explain (Bogdanov et al., 2018; Dowhan et al., 2019): (1) cytoplasmic orientation of 20% of EMDs that are net negative or neutral; (2) post-assembly dynamic changes in membrane protein topological organization; (3) co-existence of membrane proteins with dual or multiple topologies; (4) why basic amino acids generally dominant as cytoplasmic retention signals over acidic amino acids as membrane translocation signals; (5) why increasing the membrane content of anionic phospholipids does not favor cytoplasmic retention of positively charged EMDs but rather increases the membrane translocation potential of EMDs containing acidic residues.

The Charge Balance Rule (Dowhan et al., 2019) as an extension of the Positive Inside Rule was formulated to address the above shortcomings and the influence of membrane lipid composition on the dynamic topological orientation of membrane proteins (Bogdanov et al., 2018; Dowhan et al., 2019). The charge density of the membrane surface and the charge character of EMDs act in concert to determine TMD orientation at the time of initial membrane protein assembly and dynamically after initial assembly. Net zero charged PE and PC and uncharged cholesterol and glycolipids (Wikström et al., 2004, 2009; Xie et al., 2006; Vitrac et al., 2013a; Bogdanov et al., 2014), which dilute the high negative charge of anionic PG and CL, appear to dampen the translocation potential of negative residues in favor of the cytoplasmic retention potential of positive residues. Since the ratio of properly oriented to inverted LacY is dependent on the ratio of PE to PG plus CL, topological heterogeneity can arise simply through perturbations of the lipid-sensitive kinetic and thermodynamic equilibria resulting in either complete interconversion or a mixture of topological conformers.

The studies originated in *E. coli* strains lacking PE demonstrated that membrane protein-folding and dynamic post-assembly rearrangements are thermodynamically driven processes dependent on inherent lipid-protein interactions that may not require other cellular factors. These results provide a thermodynamic basis for how changes in lipid composition and post-translational modifications of proteins can change the ratio of topologically distinct populations of native and non-native conformers or explain the existence of proteins with dual or multiple topologies (Dowhan et al., 2019). Therefore, membrane protein structural organization is not static but potentially highly dynamic after initial assembly.

Several other secondary transporters also undergo topological inversions in the absence of PE or show local structural changes that affect their function (Zhang et al., 2003, 2005; Hariharan et al., 2018; Vitrac et al., 2011, 2020). Others using different approaches, have reported similar effects of lipid environment and EMD charge on MP structural organization (Bowie, 2006, 2013; Hickey and Buhr, 2011; Tunuguntla et al., 2013; McIlwain et al., 2015).

If membrane lipid composition changes have such a dramatic effect on membrane protein organization, why are PE lacking strains still viable? Thus far the changes in structure of secondary transporters do not completely inactivate the transporters but render them as facilitated rather than active transporters. The same maybe true of other proteins whose function is compromised but not completely lacking. Replacing PE with PC or other net neutral lipids does not suppress the filamentous growth phenotype or divalent metal requirement of PE-lacking strains so there are addition functions requiring specifically PE (Bogdanov et al., *2*010a). PE-lacking cells fail to induce formation of pili (Shi et al., 1993), show upregulation of the Cpx stress response system and display an increase in the outer membrane protease DegP (Mileykovskaya and Dowhan, 1997). These additional phenotypes have not been extensively investigated to determine their molecular basis.

### The Requirement for Anionic Phospholipids

Strains lacking PG and CL or CL, although viable when coupled with suppressor mutations, display several phenotypes supporting a role for wild type levels of these lipids. Phospholipids are not evenly distributed within the inner membrane of *E. coli*. Using the fluorescent dye 10-*N*-nonyl acridine orange (NAO), anionic lipid domains were first visualized at the cell poles and potential division sites of wild type and *pssA* null mutants (Mileykovskaya and Dowhan, 2000). The former domain is derived from the latter domain after cell division. NAO displays strong green fluorescence when bound to anionic lipids such as PG, PA, and CL but also shows red fluorescence when bound only to CL. The above domains show both red and green fluorescence indicating the domains are enriched in CL and possibly PG. This appears to be the case since *clsA* null strains treated with NAO during exponential growth only show green-fluorescent domains. In addition, PG and CL content increases with increasing osmolarity and in stationary phase (Romantsov et al., 2007), which is probably related to induction of *clsB* or *clsC*. In a *clsA* null strain red fluorescence was observed after reaching stationary phase in high osmolarity medium (Romantsov et al., 2007) but not during exponential growth. *E. coli* appears to have a mechanism for enriching the poles and the septum with anionic lipids. Minicells, which are derived from the cell poles, isolated from a *pgsA* null strain (thus lacking PG and CL), are enriched in PA and the minor anionic lipid *N*-acyl-PE (Mileykovskaya et al., 2009).

What role do these anionic lipids play in cell function and is there a preference for CL? Several proteins localize to the cell poles, the septal region or interact globally with the membrane surface via interaction with anionic lipids (Matsumoto et al., 2006). ProP is an osmosensory transporter whose level increases with medium osmolarity to adjust cellular levels of solutes in response to osmotic stress. ProP co-localizes with NAO fluorescence at the cell poles (Romantsov et al., 2007, 2008). This localization is considerably reduced in a *clsA* null strain and appears to be sensitive to the level of total cell CL, which also increases with medium osmolarity, independent of the level of ProP. Additionally, *clsA* null strains are sensitive to growth in high osmolarity medium. The mechanosensitive channel MscS also localizes to the cell poles in a CL-dependent manner while LacY and several mechano- and osmosensitive cell components localize to the poles in a mostly CL-independent manner (Romantsov et al., 2010). Further studies are required in a *pgsA* null strain to determine if any of the CL-independent proteins localize to the poles via anionic lipids.

The MinCDE system is required for localization of the FtsZ ring at the cell center onto which the remaining cell division proteins organize (Margolin, 2001). In the absence of the Min system the FtsZ ring localizes with the cell center and poles resulting in the budding of mini-cells from the poles. Binding of ATP to the peripheral membrane protein MinD exposes an amphitropic helix with one face being positively charged and the opposite face being hydrophobic (Zhou and Lutkenhaus, 2003). This helix partially inserts into acidic membrane domains at the cell poles. MinE binding to MinD induces ATPase activity of the latter with release of MinD from the membrane. MinC also binds to MinD and is an inhibitor of FtsZ association with the membrane. The result is that MinCD oscillate from pole to pole with significant dwell time at the poles, which restricts FtsZ localization to the cell center. In PE-lacking cells containing only anionic lipids, MinD localizes with increased dwell time to NAO fluorescent domains randomly distributed over the filamentous cell rather than at the poles (Mileykovskaya et al., 1998; Mileykovskaya and Dowhan, 2000). The preference for anionic lipids over zwitterionic PE or PC was verified in binding of Min-ATP to zwitterionic liposomes with and without PG or CL (Mileykovskaya et al., 2003). Although increased anionic lipid content in the absence of PE disrupts oscillation of MinD between anionic lipid domains (Mileykovskaya et al., 1998), complete lack of PG and CL has little effect on this oscillation presumably due to increased levels of PA and NAPE, which localize to the poles (Mileykovskaya et al., 2009).

The peripheral membrane protein DnaA is required for initiation of DNA replication at the *oriC* locus of *E. coli*. The protein is active in the ATP-bound form but after initiation of replication it is converted to the inactive ADP bound form, which does not localize to *oriC*. It was initially determined *in vitro* that anionic phospholipids induced release of ADP and facilitated ATP binding and activation of DnaA (Sekimizu and Kornberg, 1988; Crooke et al., 1992). Downregulation of expression of *pgsA* using a *lacOP* inducible promoter reduces both PG and CL levels by about 75%. Evidence for an *in vivo* anionic lipid requirement for DnaA initiation of DNA replication is supported by suppression of the lack of growth on agar plates of the *lacOP*-*pgsA* strain in the absence of inducer by a *rnhA1* null mutation (Xia and Dowhan, 1995a). This mutation allows DnaA-independent initiation of DNA replication at the alternative *oriK* site (Kogoma and von Meyenburg, 1983).

Phosphatidylglycerol can provide a conformational constraint for the conjugative *E. coli* F pilus, which is assembled from protein-phospholipid units, in which TraA pilin subunits interact with five PG’s based on cryo-electron microscopic reconstructions at 3.6–5.0 Å resolution (Costa et al., 2016). Stoichiometrically arranged PG molecules line the pilus lumen with their solvent exposed head groups directed to the interior of the pilus and the acyl chains entirely buried between subunits. PG molecules could facilitate pilus dynamics and lubricate re-insertion of pilus subunits within the inner membrane during pilus retraction/depolymerization. Alternatively, PG could lubricate naked electronegative ssDNA transport and provide directionality by maintaining appropriate electrostatics of the pilus interior. A viable *pgsA* null mutant should be used to test this hypothesis further to see whether without PG the overwhelmingly positive inside of the pilus is still able to translocate negatively charged ssDNA substrate.

Efficient insertion of proteins into the membrane and export of proteins from the cytoplasm also requires anionic phospholipids. The translocation of secreted proteins across the inner membrane of *E. coli* is significantly compromised when PG and CL levels are reduced using a promoter regulated *pgsA* strain (de Vrije et al., 1988; Kusters et al., 1991). This observation coincides with the *in vitro* data demonstrating that SecA ATPase activity, required for protein translocation, is stimulated by anionic phospholipids (Lill et al., 1990). Recent evidence implicates CL more specifically as a requirement for efficient membrane translocation of periplasmic proteins and membrane insertion of integral membrane proteins (Ryabichko et al., 2020). Both processes were severely compromised in a *clsABC* null strain. The dimeric SecYEG complex and association of SecA with the complex are required for efficient function in both processes. In the *clsABC* null strain SecYEG was mostly monomeric with minimal associated SecA supporting a more specific requirement for CL over PG.

Other anionic phospholipids appear to partially replace CL resulting in normal cell growth under optimal conditions. However, lack of CL under stressed conditions should be further investigated since CL-lacking strains display several phenotypes that might compromise growth under less optimal conditions, as summarized below (Rowlett et al., 2017). Cls expression and CL biosynthesis are highly regulated and modulated during abiotic stress in both Gram-positive and Gram-negative microorganisms (Luevano-Martinez and Kowaltowski, 2015), which respond to many abiotic stressors by increasing the proportion of CL. Expression of *clsA* is increased several-fold (Heber and Tropp, 1991) and the amount of CL increases as PE decreases irrespective to how osmotic stress is imposed (Tsatskis et al., 2005). Stepwise increase in CL content of the bacterial membrane due to growth in media of increasing osmolarity results in conformational changes of ProP, which transports the osmoprotectants proline and glycine betaine (Tsatskis et al., 2005). Due to its unique conformational properties, CL could be considered a stress responsive molecule required to adjust physical properties of bacterial membrane (Luevano-Martinez and Kowaltowski, 2015).

### Comparing PE-Lacking and CL-Lacking Cells

As already noted above, lack of PE or CL is not lethal under specific growth conditions, but the cells are not normal. In fact, a systematic scanning of cell morphology and cell function revealed multiple deficiencies in these mutants (Rowlett et al., 2017). Elimination of either lipid results in alteration in cell morphology and structural organization of the cell envelope, the ability to form biofilms, the tolerance to environmental stress, and reduced cellular robustness. CL-lacking cell growth characteristics were largely the same as wild type cells. However, cell viability was compromised in stationary phase, which may be related to the requirement for increased CL levels in stationary phase. Transfer of cells to defined minimal medium resulted a long lag in initiation of growth for PE-lacking cells and a significant increase in the doubling time for CL-lacking strains. For both mutants irrespective of growth medium, cell length heterogeneity was greater than for wild type cells. Envelope ultrastructure was disrupted in both mutants but greater for the PE-lacking cells. The periplasmic width was greatly increased, and considerable amounts of electron dense material accumulated in PE-lacking cells. In both mutants there was considerable extracellular material indicative of cell lysis or loss of envelope material. CL-lacking cells displayed twisted and spiral cell envelope structures in minimal medium. The outer membrane of PE-lacking cells is leaky to periplasmic macromolecules as evidenced by extracellular periplasmic RNAse (Wikström et al., 2004). This may be due to the lack of PE as the major lipid of the inner leaflet of the outer membrane, the lack of decoration of LPS by ethanolamine-P derived from PE (Schnaitman and Klena, 1993; Kanipes et al., 2001), or the significant shortening of the *O*-antigen repeats of the outer leaflet LPS (Rowlett et al., 2017). On the other hand, CL-lacking cells have an extended *O*-antigen repeat. Surface adhesion and biofilm formation was also adversely affected in cells lacking PE or CL. Tolerance to both osmotic and oxidative stress was also significantly reduced as PE levels were reduced in a dose dependent manner. Lack of CL also reduced tolerance to osmotic stress but increased tolerance to oxidative stress. The above results clearly demonstrate that large adverse changes occur in cell structure and physiology when the evolutionary determined optimal membrane lipid composition is perturbed.

## Regulation of Phospholipid Composition

*E. coli* membrane phospholipid composition and content remain within a narrow range under a broad range of growth conditions (Dowhan, 1997b). Small variation in phospholipid composition occurs with growth conditions and between strains, but composition remains at 70–80% zwitterionic PE and 20–30% anionic PG, CL, and phospholipid precursors. Except for the reversible formation of CL from PG by Cls’s, all steps in the pathway are irreversible under physiological conditions. PG ranges from 20–25% and CL 5–10% with this variability due to increased conversion of PG to CL as cell growth slows, media osmolarity increases or cells approach stationary phase.

How the ratio of zwitterionic to anionic phospholipid is regulated is still not well understood, although it must occur at the branchpoint in the biosynthetic pathway following the formation of CDP-DAG. A conditionally lethal *cdsA* mutant accumulates PA mostly at the expense of PG and CL rather than PE suggesting that affinity for CDP-DAG may be higher for the PssA than for PgsA (Ganong and Raetz, 1982). Massive 150-fold overproduction of PssA (Ohta et al., 1981a) resulted in no change in the above ratio of PE to PG plus CL while a 40-fold overproduction of PgpA (Ohta et al., 1981b) resulted in a change in the PE to anionic lipid ratio from 75/25 to 65/35 but nowhere in proportion to enzyme overproduction. A possible reason for lack of a response to overproduction of PssA lies in the peripheral membrane association of the enzyme and its membrane association dependent on binding to its lipid substrate CDP-DAG or anionic lipids (Carman and Dowhan, 1979; Louie and Dowhan, 1980). CDP-DAG remains constant and at barely detectible levels while an increase in the anionic lipid would increase PssA membrane association resulting in normalizing the ration of PE to PG plus CL. The overall control mechanism is supported by replacement of the *E. coli* PssA with the integral membrane enzyme from *B. subtilis*. In this case the level of PE increased with increasing amounts of the latter enzyme (Matsumoto, 1997).

Successful expression and function of the Kennedy Pathway enzymes from *E. coli* inside of large unilamellar lipid vesicles (Blanken et al., 2020) composed of controlled ratios of PG to PE largely supports regulation of PssA activity by PG content. PE synthesis was dependent on the PG content of the liposomes independent of the level of expression of the PG-synthesizing branch. This result indicates that the regulatory mechanism is not solely dependent on competition between the two branches of the pathway for CDP-DAG but relies on the association of PssA with anionic lipids as predicted from the earlier reconstitution experiments (Louie and Dowhan, 1980; Louie et al., 1986).

PlsB initiates phospholipid synthesis utilizing G3P as a substrate. PlsB activity is coordinated with other macromolecular synthesis via guanosine pentaphosphate (ppGpp), and the *plsB* gene is induced by the σ^E^ stress response regulator (Wahl et al., 2011; Yao and Rock, 2013). Thus, PlsB levels respond to coordinate down regulation of macromolecular synthesis under nutrient limiting conditions via ppGpp inhibition, and the enzyme level is increased in response to increases in envelope stress. Since PlsB produces a new PA while DgkA salvages PA using existing pools of DAG, these enzymes would be expected to be regulated in a reciprocal manner by σ^E^ and ppGpp, which in fact is the case (Wahl et al., 2011). PlsB activity is promoted by PG implying that PG is involved in a positive feedback loop that produces PA and thus all membrane lipids (Ishinaga et al., 1976; Scheideler and Bell, 1989).

The high Km PlsB mutant is dependent on glycerol for growth and phospholipid synthesis (McIntyre et al., 1977). Removal of glycerol from the medium results in immediate cessation in phospholipid synthesis and cell growth. However, membrane protein synthesis continues until the ratio of membrane protein to phospholipid increases about twofold. Re-supply of glycerol initiates new phospholipid synthesis immediately but membrane protein synthesis and cell growth lag until the membrane protein to phospholipid ratio returns to normal levels. Therefore, the cell senses and regulates this ratio maintaining membrane capacity for protein well below the maximum.

Overproduction of PlsB results in filamentation and massive accumulation of intracellular tubular membrane structures (Wilkison et al., 1986). The isolated structures were highly enriched in PlsB, contained an increased protein to lipid ratio compared to the cell membrane and a wild type phospholipid composition. Similar results have been observed with overproduction of other membrane proteins except that in many cases CL levels were also elevated (Arechaga, 2013; Jamin et al., 2018). Depletion of CL in a *clsABC* triple null mutant disrupts formation of these intracytoplasmic membranes in a strain overproducing F-ATPase subunit *b* (Carranza et al., 2017). This result implies a physical requirement supplied by CL in forming these intracellular structures. However, clear understanding at the molecular level of how cells coordinate phospholipid synthesis, membrane protein levels and cell growth are not well understood.

## Membrane Phospholipid Asymmetry

Most if not all biological membranes display an asymmetric distribution of lipid species on each side of the bilayer (Marquardt et al., 2015). Such asymmetry in biological membranes is entropically disfavored and expected to be in a non-equilibrium thermodynamic state. This non-equilibrium situation appears to be maintained by membrane proteins known as phospholipid translocators (flippases and floppases), which use ATP to mediate the net transfer of specific phospholipids from one leaflet of a membrane to the other. Scramblases equilibrate lipids in both directions across the membrane and can abolish lipid asymmetry. Several candidate flippases have been identified in eukaryotes that catalyze translocation of different classes of lipids (Pomorski and Menon, 2006; Sharom, 2011; Lopez-Marques et al., 2014).

MsbA is the only bacterial phospholipid flippase identified in *E. coli*. Conditionally lethal *msbA* mutants accumulate phospholipids and LPS in the cytoplasmic leaflet of the inner membrane upon a shift to non-permissive conditions (Doerrler et al., 2004). Unfortunately, MsbA has never been demonstrated to promote translocation of phospholipids *in vitro*. Thus, either MsbA alone is necessary but not sufficient for distribution of phospholipids among lipid monolayers *in vitro* or unknown accessory proteins are required for MsbA to perform efficient phospholipid translocation. It is also possible that MsbA is involved only in LPS lipid A transport but not responsible for phospholipid distribution within the cell envelope.

Eukaryotic cell plasma membrane lipid asymmetry is well established, and loss of asymmetry is associated with an array of cellular malfunctions (Doktorova et al., 2020). However, the importance of lipid asymmetry is not fully understood especially in bacteria. In Gram-positive *Bacillus megaterium* 68% of the PE resides in the cytoplasmic leaflet of the single cell membrane (Rothman and Kennedy, 1977a). The outer membrane lipid bilayer of *E. coli* is asymmetric with LPS exclusively in the outer leaflet and primarily PE in the inner leaflet (Ganong et al., 1980). Determination of the transbilayer lipid distribution of the inner membrane of Gram-negative bacteria has been complicated by contamination with the outer membrane enrichment in PE.

Recent isolation of uniformly oriented inside-out inner membrane vesicles of *E. coli* and *Yersinia pseudotuberculosis* essentially free of outer membrane made possible the determination of the distribution of PE within the inner membrane lipid bilayer of these bacteria (Bogdanov et al., 2020). Inside-out inner membrane vesicles were treated sequentially with a membrane impermeable primary amine probe followed by a membrane permeable amine probe. The probes had different chromogenic properties so that after solvent extraction of the vesicle lipid derivatives, spectral determination was used to determine the amounts of periplasmic and cytoplasmic leaflet PE. The cytoplasmic/periplasmic leaflet distribution of PE in these two Gram-negative bacteria is 75/25 using 3 independent methods of determination (Bogdanov et al., 2020). Although *E. coli* PE is about 75% of total phospholipid, the outer membrane periplasmic leaflet is about 90% PE (Ganong et al., 1980). Thus, the inner membrane bilayer phospholipid is about 65% PE (Ganong et al., 1980; Bogdanov et al., 2020). The remaining phospholipids are anionic PG, CL, and phospholipid precursors. Currently the transmembrane distribution of the individual anionic phospholipids is not known. Using these numbers, the periplasmic leaflet of the inner membrane is composed of a near equal amount of zwitterionic and anionic phospholipid and the cytoplasmic leaflet contains a 75/25 enrichment of PE over anionic phospholipids.

Although the inner membrane bilayer distribution of PE is similar to that reported for *B. megaterium* (Rothman and Kennedy, 1977a), there is a major difference in the appearance of newly synthesized PE between these organisms. In *B. megaterium*, which contains a single lipid bilayer, newly synthesized PE appears in the cytoplasmic leaflet followed by distribution to the outer leaflet (Rothman and Kennedy, 1977b). In *E. coli* both newly synthesized PE and its precursor PS (traced in a *psd2* temperature sensitive mutant) first appear in the periplasmic leaflet followed by distribution to the cytoplasmic leaflet (Langley et al., 1982; Bogdanov et al., 2020). This is quite surprising in that PssA is a peripheral membrane protein that associates with the cytoplasmic surface of the inner membrane (Louie and Dowhan, 1980; Louie et al., 1986). Interestingly, PS synthesis initiated on the luminal leaflet of large unilamellar lipid vesicles is also immediately translocated to the outer leaflet, where it is detected by a bulky PS-specific fluorescent reporter probe (Blanken et al., 2020). Since PE is required in both the outer and inner membrane, initial appear in the periplasmic leaflet of the inner membrane may allow efficient distribution to both membranes. Upon induction of PE synthesis in a PE-lacking strain of *E. coli*, PE also initially appears in the periplasmic leaflet of the inner membrane (Bogdanov et al., 2020). As the PE content increases from 0 to 75%, the distribution between the inner membrane leaflets approaches wild type levels. Inducement of filamentation of wild type *E. coli* also reverses the distribution of PE to favor the periplasmic leaflet at steady state.

How PE distributes dynamically as its level is increased through regulated synthesis of PE in cells initially lacking PE coupled with mutants in CL synthesis was used to determine how PE and CL affect lipid order in the bilayer. Using fluorescent sensors of lipid order, it was determined that the increase in packing order driven by PE is countered by the increase in disorder driven by CL (Bogdanov et al., 2020). This *in vivo* affect mimics the results of studies done in artificial membranes free of proteins. The driving force for inner membrane lipid asymmetry can arise from the packing requirements imposed upon the system by the opposing forces of two negatively curved phospholipids in both leaflets. Therefore, percentage and localization of PE and CL appears to be adjusted to satisfy lipid packing requirements in order to maintain a stable bilayer and proper membrane morphology.

**FIGURE 4:**
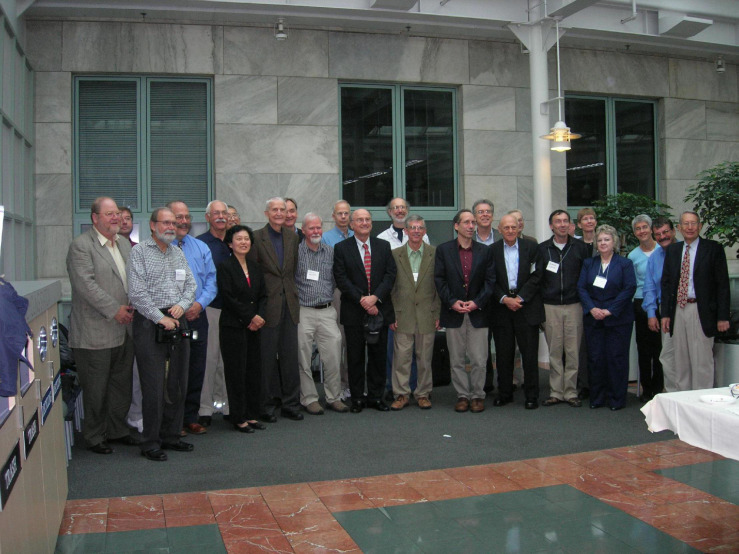
The Kennedy “Clan” on the occasion of his 90th birthday. Eugene Kennedy (1919–2011) is 3rd from the left in the front row. The gathering was in October 2009 at Harvard Medical School. Pictured are former graduate students, postdoctoral fellows and scientific associates of Eugene Kennedy. Figure and legend reprinted by permission from Elsevier (Dowhan, 2013): Copyright 2013.

Clearly phospholipids are in constant flux within the inner membrane and between the outer and inner membranes (Langley et al., 1982; Bogdanov et al., 2020) in Gram-negative bacteria. This flux may be necessary to maintain entropically disfavored asymmetric transmembrane arrangement of lipids in both membranes by continuous retrograde transport of phospholipids from the outer to the inner membrane (Malinverni and Silhavy, 2009; Powers and Trent, 2018) and anterograde trafficking back to outer membrane (Hughes et al., 2019; Grimm et al., 2020). Membrane growth and continuous emergence of lipids in the periplasmic leaflet of the inner membrane for translocation to the outer membrane requires a constant supply of phospholipids in the cytosolic leaflet. Thus, asymmetry within the inner membrane may be regulated metabolically driven by insertion of PE in the periplasmic leaflet of the inner membrane followed by a balance between transfer to the outer membrane and the cytoplasmic leaflet of the inner membrane, which would relieve the lateral pressure within the periplasmic monolayer (Bogdanov et al., 2020). The shape difference between rod-shaped and filamentous cells may perturb the normal rates of distribution of PE within the cell envelope suggesting that PE distribution may facilitate or result from changes in bacterial shape. Indeed, the gradual changes in distribution of PE/CL amounts between the inner membrane leaflets during *de novo* PE biosynthesis in *E. coli* cells initially lacking PE coincides with progressive reduction of cell size as PE is progressively accumulated and CL is removed from the cytoplasmic leaflet of the inner membrane (Bogdanov et al., 2020). See Figure 4 for Kennedy associates who attended his 90th birthday celebration.

## Summary and Perspectives

Prior to laying the foundation for bacterial phospholipid metabolism and enzymology in the 1960’s and 1970’s, Eugene Kennedy did the same for the somatic cell phospholipid field (Dowhan, 2013). Dennis Vance, Jean Vance, Claudia Kent, and Suzanne Jackowski extended Kennedy’s work in somatic cells by detailing the genetics and enzymology. Susan Henry and George Carman defined the genetics and biochemistry of phospholipid metabolism in *S. cerevisiae*. There were other major contributors to the microbial phospholipid field. Roy Vagelos, John Cronan, Robert Bell, and Charles Rock defined microbial fatty acid synthesis and the synthesis of PA. The Dutch group initially formed by Laurens van Deenen made major contributions to lipid enzymology and metabolism. Japanese laboratories of Isao Shibuya, Kouji Matsumoto, and Akinori Ohta not only contributed to the *E. coli* phospholipid field but extended studies to Gram-positive bacteria. Many of Kennedy’s trainees from the late 1960’s to 1970’s went on to make seminal contributions in areas related to cell membranes and lipids as have many of their trainees: Chris Raetz in phospholipid genetics and Lipid A studies in Gram-negative bacteria; William Wickner in membrane protein assembly; Edward Dennis in phospholipases and lipid second messengers; Carlos Hirschberg in mammalian nucleotide sugar transporters; Dennis Voelker in *S. cerevisiae* and lung phospholipid metabolism; and Nobel Laureate James Rothman in intracellular trafficking of secreted proteins.

There is still much to understand and study. Methods for determining anionic lipid bilayer asymmetry are required in order to develop a full picture of the importance of this asymmetry in microbial physiology. How is bilayer lipid asymmetry generated and controlled? What processes are responsible for transmembrane and intramembrane movement of phospholipids in Gram-negative bacteria? How is phospholipid synthesis and composition regulated and coordinated with other macromolecular synthesis? Thus, identification of the mechanism (flippase-free or flippase-guided) controlling the asymmetric distribution of lipids within the cell envelop of Gram-negative bacterial should be a high priority goal.

Given the defects in mutants lacking the major phospholipid classes, metabolic reactions with mechanisms unique to bacteria need to be exploited as antimicrobial targets. In particular PssA and ClsA fall into this category. Clearly, lipid and lipid enzyme inspired therapeutics and chemical targeting of the bacterial lipid synthesis is in its infancy, and it remains to be seen whether the significant challenges in protein biochemistry and drug design can be overcome to target essential lipid enzymes. Such studies will be aided by high resolution structural studies of phospholipid biosynthetic enzymes. Significant effort should be made to identify potential contribution of genes that are not exclusively engaged in antibiotic resistance but are involved in regulation of cell envelope synthesis, morphology and remodeling.

Many phenotypes have been observed for the current set of null mutants that need more detailed studies to expand the understanding of the role of membrane lipids in specific cell functions. Information on the biosynthesis of phospholipids in bacteria has been derived from a relatively small set of easily culturable bacteria. Studies in many pathological microorganisms are complicated due to defining appropriate growth conditions. Very few studies of bacterial phospholipid metabolism and function have been carried out under anaerobic conditions, which may more closely mimic conditions for both symbiotic and pathological presence of bacteria in mammalian systems. Given the complexity and challenges of defining functions of lipids in multi-organelle somatic cells, clues from bacterial studies will provide valuable information on the role of lipids in all cell types.

## Author Contributions

All authors listed have made a substantial, direct and intellectual contribution to the work, and approved it for publication.

## Conflict of Interest

The authors declare that the research was conducted in the absence of any commercial or financial relationships that could be construed as a potential conflict of interest.
